# Evaluating cow identification reliability of a camera-based locomotion and body condition scoring system in dairy cows

**DOI:** 10.3168/jdsc.2024-0659

**Published:** 2024-12-12

**Authors:** D. Swartz, E. Shepley, G. Cramer

**Affiliations:** Department of Veterinary Population Medicine, University of Minnesota, St. Paul, MN 55108

## Abstract

Dairy farms are growing in size, while the total number of farms is decreasing. As herd sizes grow, technology is increasingly adopted for monitoring and managing cows. However, for technology to replace subjective and labor-intensive tasks, accurate technologies are needed. Camera-based systems are being explored for improving management, particularly around lameness and body condition scoring. A key requirement a camera-based technology is its ability to accurately identify the animal being observed. The primary objective of this study was to evaluate the cow identification (ID) accuracy of a camera-based technology that locomotion scores and body condition scores dairy cattle. Secondary objectives were to determine the number of days required for initial identification and evaluate the frequency of animal recognition. Two dairy sites in Minnesota were visited twice and a total of 105 cows were enrolled from site A (n = 40) and site B (n = 65). Each cow had their cow ID and radio frequency identification recorded. All cows that had been in the pen for 5 or less days were enrolled in the study and had a combination of colors, letters, and numbers painted on their rumps as unique identifiers (paint ID; PID). The video feed from the camera-based technology was observed daily from the company website for 7 d. A cow was recorded as correctly identified if the website had an uploaded video for a cow's ID that matched its PID. Successful identification was defined as the proportion of cow ID for which video was uploaded to the user platform within 7 d from the start of the study, regardless of accuracy. Correct identification was subsequently calculated as the proportion of these successfully identified cows that had a PID corresponding to their PID. The days under the camera were calculated by including the time cows would have been exposed to the camera before our 7-d observation period. Additionally, days identified reflect the total number of days that cows were identified (correctly or incorrectly) and scored during the observation period. Of the 103 cows enrolled, 87 (84.5%; 95% CI: 76%–91%) of cows were successfully identified during the 7-d study period. One cow from those 87 was incorrectly identified, resulting in a correct identification of 98.9% (95% CI: 94%–100%). Of the 86 correctly identified cows, all cows were observed between days 4 and 11 under the camera. Of the cows identified, they were identified 1 to 7 times. This technology accurately identifies cows, but 16 cows were not initially identified and ended with a minimum and maximum of 7 and 11 d under the camera, respectively. To allow management decisions to be made early in lactation or for new cows entering the herd, the technology will need to accurately identify all cows within the first week of being exposed to the camera.

## Highlights

• The technology was 98.9% accurate in identifying cows.
• All but 16 cows (16%) were identified between 4 and 11 days of exposure to the camera.
• The number of days until cows were first identified varied by farm site.

## Graphical Abstract

**Figure:**
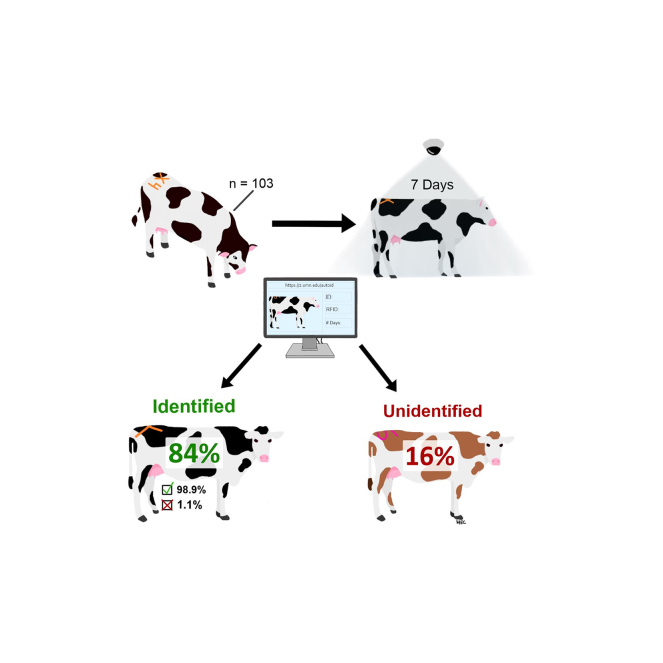
**Summary:** Dairy farms are growing in herd size, requiring management efficiency improvements. One method of improving management efficiency is through the use of camera-based technology. A technology that scores for lameness and body condition was evaluated for its cow identification accuracy and days until recognition. The technology was 98.9% accurate, with 84% of cows identified after being exposed to the camera for 4 to 11 days.

In the United States, the number of dairy farms is decreasing and the average herd size is increasing (USDA, 2023). With herd sizes increasing, the operation of monitoring and managing cows is growing in complexity, and technology can help with the changes in management-required improvements (Edwards et al., 2014; Bewley, 2016). Precision technology can assist in multiple areas, such as reproduction, milking, and calf management (Palma-Molina et al., 2023). Furthermore, Gargiulo et al. (2018) showed that farms with more than 500 cows had greater precision technology adoption on individual farms than smaller herds, and that farmers expected that the technologies with the highest rate of adoption would be automatic estrus detection systems, automatic sorting gates, and automated mastitis detection tools.

Camera-based approaches are being used for monitoring health and disease detection (Beaver and Rutter, 2023). A requirement of a camera-based technology's efficacy is its ability to accurately identify the observed animal. Specific to cow identification (**ID**) technology, Shen et al. (2020) designed an automatic algorithm for identifying cows with side-images and obtained an accuracy of 96.7%, where identification errors mainly occurred on cows whose main color is black, cows with small individual differences, and blurry images. A recently developed and commercially available camera-based technology (CattleEye Ltd., Belfast, United Kingdom) that uses artificial intelligence-driven algorithms to score dairy cow gait from overhead video recording has been validated for locomotion scoring (Anagnostopoulos et al., 2023) and body condition scoring (Siachos et al., 2024). The ID accuracy of this technology has not been evaluated, and a recent observational study showed a wide range of scores between cows with hoof lesions compared with those without (Swartz et al., 2024). One possible explanation for this variation is ID errors; therefore, it is necessary to formally evaluate the ID accuracy of the camera-based technology. The primary objective of this study was to assess the successful and correct identification of cows by the camera system. The secondary objectives were to determine the number of days required for initial identification and evaluate the weekly ID frequency.

The protocols involving animals in this study were approved by the University of Minnesota Institutional Animal Care and Use Committee (St. Paul, MN; protocol number 2206-40148A).

One dairy operation with 2 sites in Minnesota that used a commercially available, camera-based technology (AUTO, Cattle Eye Ltd., Belfast, United Kingdom) was visited twice per site with cows enrolled for each visit. All cows calved at site B and first-lactation animals were moved to site A at ∼11 to 37 DIM based on health status. The camera-based technology uses artificial intelligence-driven algorithms to score dairy cow gait (Anagnostopoulos et al., 2023) and body condition (Siachos et al., 2024) from overhead video recording located in the return alley from the milking parlor. This system identifies cows passing under the camera using a combination of radio frequency identification (**RFID**) data obtained from the cows' collar tags and visual recognition of the cow by the system's software. Both sites used the same RFID and reader technology (Nedap Agri, Groenlo, the Netherlands; GEA Farm Technologies, Düsseldorf, Germany). Site A had 2 return alleys from its parlor, with each alley having one camera installed, and site B had 1 return alley.

Animals from the sites were eligible for enrollment if they were Holsteins and their days in the pen were 5 or less at the time of enrollment. Sample size was calculated using a population proportion sample size calculator (Dhand and Khatkar, 2014). The margin of error was set at 5%, to allow for a 5% difference between the sample results and with a 95% CI and an estimated sample accuracy of 95%. A sample size of 73 was calculated. With this being conducted on 2 sites from one commercial dairy, the sample size was inflated to 100. A total of 105 cows were enrolled over 2 visits between June and August of 2023 with 40 from site A and 65 from site B.

To enroll cows, an RFID reader (AWR300 Stick Reader, Allflex, DFW Airport, TX) was used to link the cow's RFID with her visual ID. Livestock paint (QUIKSHOT, LA-CO, Elk Grove Village, IL) was used to paint the left and right sides of the rump with unique number and letter combinations using orange and pink paint colors (Figure 1). The combination of colors, letters, and numbers used for each cow (paint ID; **PID**) was recorded. In addition, after painting each cow, a picture was obtained using a cellular device (iPhone 14, Apple, Cupertino, CA).

**Figure 1:**
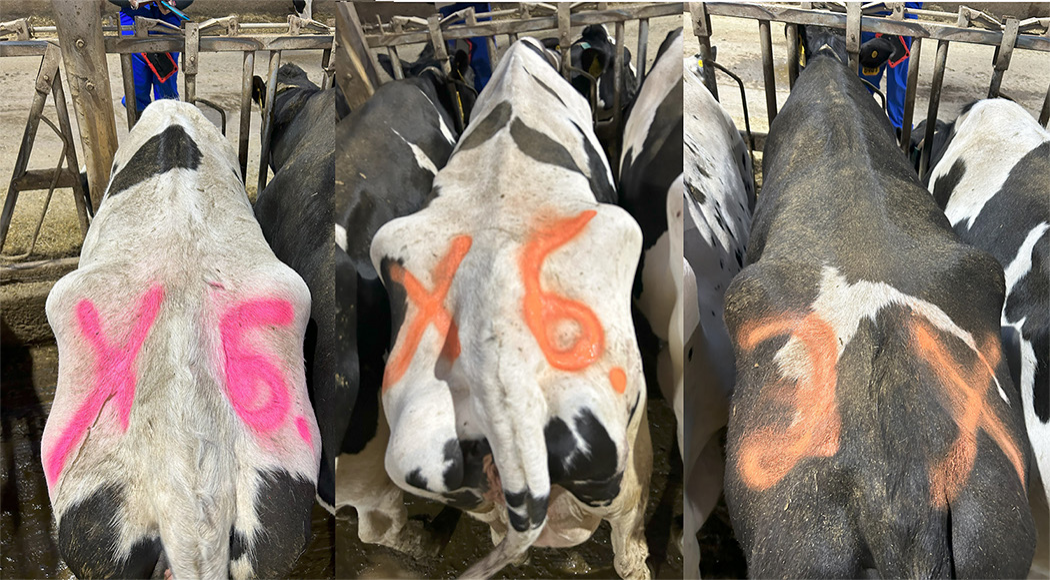
Examples of the unique number and letter combinations painted on the rump of the cow with orange and pink paint. From left to right, X 6 (pink), X 6 (orange), and 2 X (orange).

Site A and site B had the camera technology monitoring their herd between 13:00 and 21:00 and between 17:30 and 00:30, respectively. Videos of cows and their scores were pushed to the technology website (cattleeeye.com; CattleEye Inc., Belfast, Ireland) for users to access. To determine if the technology accurately identified the cow ID, the video footage captured by the camera technology was viewed by author DS on the company's website once daily between 12:00 and 20:00. This time was chosen because it was before the time that the next day's scores were uploaded. The procedure was repeated for a total of 7 consecutive days following enrollment. During each day of observation, the following items were recorded: (1) whether the cow ID was identified by the camera system and (2) whether the cow ID recorded matched the PID.

At the end of the study, cow ID and RFID were extracted from the farms' herd management software (DairyComp 305, Valley Agricultural Software, Tulare, CA) and compared with the cow ID and RFID recorded by study personnel at each site. This was used to identify cows with different RFID tags than those that were registered to them in the herd management software. Cows with mismatched RFID were removed from the analysis.

Each day, cow ID was recorded as correctly identified if the video for a cow's ID matched its PID. Successful identification was defined as the proportion of cow ID with video uploaded to the user platform within 7 d from the start of the study, regardless of being correct. Correct identification was then calculated as the proportion of these successfully identified cow ID that accurately matched the corresponding PID. Additionally, the 95% CI were computed using the Clopper–Pearson exact method. The first identified study day represents the day of the study (d 1 to 7) relative to each enrollment visit (d 1) when the website had an uploaded video for a cow ID. Our inclusion criteria considered the possibility that some cows may have passed under the camera up to 5 d before study enrollment, as they were moved into pens that were monitored before the visit dates. Because only lactating pens were exposed to the camera, this pre-enrollment exposure could have facilitated early recognition by the AUTO system before our formal study observations began. As such, the total days a cow was exposed to the camera was used to calculate the days under the camera, which indicates the total days a cow passed under the camera before identification. Days identified reflects the total days that cows were identified and scored during the 7-d observation period. Data and code are available at Data Repository for the University of Minnesota (see Notes).

Before the calculations for our objectives, 2 cows from site A had mismatched RFIDs and were removed. Of the 103 remaining cows, 38 first lactation cows from site A had an average (SD) DIM and days exposed to the camera of 17.5 (4.4) and 2.2 (1.8), respectively. Site B contributed 25 first-lactation, 19 second-lactation, 11 third-lactation, and 10 fourth-or-greater lactation cows with an average (SD) DIM and days exposed to the camera of 2.5 (1.1). Site B had an error with uploading the video data for 34 cows, resulting in d 3 and 4 being exported on the same day and causing d 3 to be unusable.

A total of 87 (84.5%; 95% CI: 76%–91%) of the total 103 cows had successful identification, with 31/38 (81.6%; 95% CI: 66%–92%) and 56/65 (86.2%; 95% CI: 75%–93%) coming from site A and site B, respectively. Of the cows successfully identified, one cow from site B was incorrectly identified from d 2 forward, resulting in a correct identification of 86/87 (98.9%; 95% CI: 94%–100%) for AUTO.

Of the 86 correctly identified cows, 10 cows were identified on the first day of the study, all of which had been exposed to the camera for either 4 (2 cows) or 5 d (8 cows) at the time of enrollment. For the 76 cows that were not identified on the first study day, they were correctly identified between days 4 and 11 under the camera (Table 1). The 86 cows were correctly identified for a range of 1 to 7 d during the study observation period but did not have identifications every day (Figure 2). Of the 16 cows not identified, the minimum time exposed to the camera was 7 d, and the maximum was 11 d.

**Table 1:** Distribution of the days under camera in frequency (percent) for cows that were correctly identified after the first study day

Days under camera^1^	Cows correctly identified after the first study day	
	Site A (n = 28)	Site B (n = 48)
4	0 (0)	7 (14.6)
5	4 (14.3)	11 (22.9)
6	7 (25.0)	18 (37.5)
7	9 (32.1)	6 (12.5)
8	6 (21.4)	3 (6.3)
9	1 (3.6)	3 (6.3)
11	1 (3.6)	0 (0)

1 Number of days the cow would have passed under the camera when it had its first identified day on the study.

**Figure 2:**
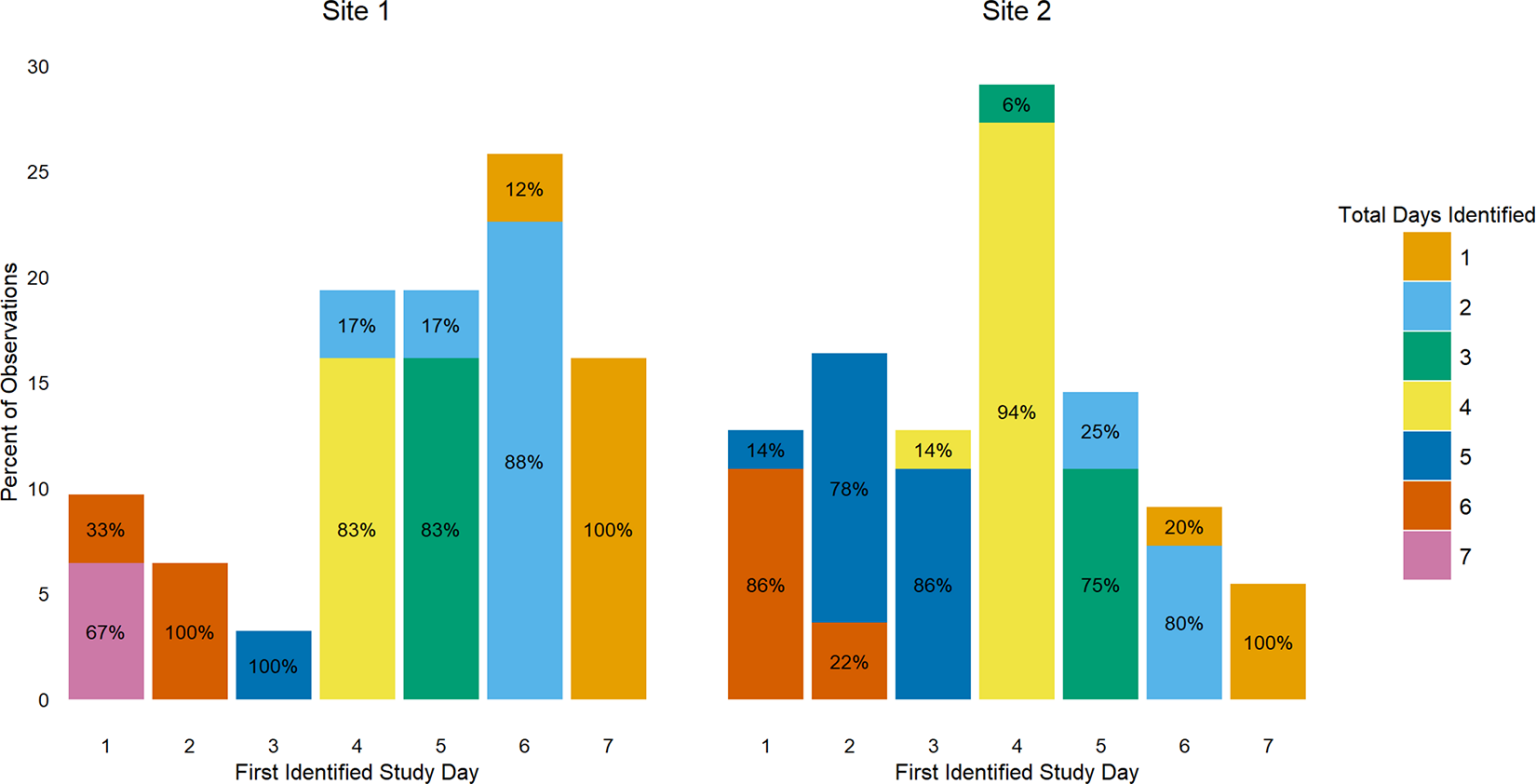
Stacked bar graph showing the percentage of cows identified for the first time on each study day at each site, with the color of the bar showing the total number of days those cows were identified.

The primary objective of this study was to evaluate the accuracy of cow recognition of a camera-based technology that performs locomotion scoring (Anagnostopoulos et al., 2023) and body condition scoring (Siachos et al., 2024). In addition, the secondary objectives were to assess the time required for identification to occur and evaluate the total days of identification during a 7-d study period. Overall, 98.9% of the cows that were identified were accurately identified by d 7 of the study, which is compatible with the 96.7% accuracy seen by Shen et al., (2020). However, 16% of cows were not identified by the end of the 7-d period for reasons that remain unclear, limiting the technology's effectiveness during early lactation and for new cows entering the herd. It is unclear whether the effectiveness is a result of on-farm conditions or limitations of the technology itself; potential reasons could include cows bypassing the camera, traffic congestion in the barn, or failure to be read by the on-farm RFID reader. The accuracy of Shen et al. (2020) was a result of a convolution neural network that used a training set and validation set. This study was a result of field application and demonstrates the technology's performance in real-world conditions. Additionally, the current study did not involve manual processing of the videos, whereas a developmental project by Shen et al. (2020) performed manual processing that removed frames that had cows overlapping.

The correctly identified cows were identified between 4 and 11 d passing under the camera system, whereas the unidentified cows ended the study with a range of 7 to 11 d passing under the camera. The unidentified cows present an issue for monitoring locomotion scores and BCS in early lactation. Given that most cows are not routinely monitored in the dry period and lameness in the week before calving is related to lameness later in lactation (Daros et al., 2019), it is therefore important for the technology to be able to identify cows quickly after calving. Additionally, the loss of BCS between calving and peak lactation is associated with compromised reproduction performance (López-Gatius et al., 2003; Chebel et al., 2018). Therefore, there is a need for this technology to reduce the number of days until identification to capture lameness and BCS measures in the first week of lactation. There may be potential in reducing the number of days until identification if the technology were to monitor cows multiple times per day as opposed to the once-daily approach in our study's sites. Additionally, for cows that are known to the camera from their previous lactation, it would be expected that they may be identified sooner compared with cows never identified by the camera, as their hide patterning would be consistent.

To improve the recognition of objects in videos, it is necessary to use methods that balance speed and accuracy (Bhende et al., 2022) to decrease processing time for large herds. These methods must also maintain accuracy under varying lighting conditions (Murthy et al., 2020) and handle dynamic scenes effectively (Jha et al., 2021). Throughout the day, lighting changes on farms may hinder the ability to confidently recognize differences between primarily black or similarly patterned Holsteins. Additionally, congestion in return alleys may make it difficult for the technology to recognize and identify a single cow. Addressing these challenges may lead to more reliable outputs and quicker identification.

A limitation of the study is that the comparison of the RFID recorded in the herd management software and RFID from the cow's collar was conducted after enrollment. This improper pairing of RFID reduced our cow numbers by 2; thus, future studies should verify the cow ID and RFID pairings before starting a study. Additionally, the observation period only lasted 7 d and did not follow each cow ID until recognition, which would have provided a true measure of accuracy and days until recognition. Furthermore, although the absence of statistical analysis could be seen as a limitation, it was intentional in this study. The primary goal was to provide a descriptive assessment of the technology's performance rather than test specific hypotheses.

Our study gives confidence in the technology's capability for accurate cow identification. However, the fact that 16 cows were not identified during the study period limits the utility of the technology for monitoring BCS and lameness around calving and for new cows entering the herd. Of the cows identified during our study, the range of days under the camera was 4 to 11 d, and at least 7 to 11 d for cows not identified. If the technology is to be used to capture measures of BCS and lameness around calving or for when new cows enter the herd, then the number of days required for identification must be reduced.
